# Multi-Factor Aging Mechanism and Multi-Parameter Synergistic Lifetime Prediction of HTV Silicone Rubber Composite Insulators

**DOI:** 10.3390/polym18141701

**Published:** 2026-07-10

**Authors:** Haocheng Liu, Bowen Wang, Zhiyao Fu, Kai Ning, Zhuan Jin, Haining Wang, Zhenglong Jiang

**Affiliations:** State Key Laboratory of Disaster Prevention & Reduction for Power Grid, Disaster Prevention and Reduction Center of State Grid Hunan Electric Power Co., Ltd., Changsha 410100, Chinaningkai@hnu.edu.cn (K.N.);

**Keywords:** HTV silicone rubber composite insulator, multi-factor aging, aging mechanism, multi-parameter synergy, lifetime prediction

## Abstract

The long-term aging of HTV silicone rubber composite insulators under complex environments severely threatens power transmission line reliability. Traditional single/dual-parameter aging evaluation and lifetime prediction methods have low accuracy and poor generalization because they generally ignore multi-factor synergistic effects, pollution accumulation and electrical erosion. In this work, we systematically studied the aging characteristics of HTV silicone rubber under UV radiation, humidity, salt/ash contamination and their combined effects via accelerated aging tests and field sample verification, quantitatively analyzed the evolution of key mechanical, electrical and hydrophobic properties, and revealed the multi-factor synergistic aging mechanism from a micro–macro perspective with SEM, XPS and FTIR. The acceleration factor of the comprehensive accelerated-aging test was calculated as 43.8 through field-performance matching between 2000 h laboratory aging and 10-year field aging. We further established a “physical-pollution-electrical” three-dimensional evaluation system and constructed a multi-parameter synergistic lifetime prediction model through Pearson correlation analysis, VIF diagnosis and multiple stepwise regression. Compared with traditional dual-parameter models, the proposed model integrates physically interpretable degradation, contamination and electrical-stress indicators, thereby improving both prediction accuracy and engineering traceability. The model has 33% higher accuracy than traditional dual-parameter models, with average prediction errors of 0.55 years (same-manufacturer) and 1.0 years (cross-manufacturer). Five-fold cross-validation gives an MAE of 0.62 ± 0.08 years, and independent testing shows that most validation samples fall within the 95% prediction intervals, confirming favorable applicability within the present validation scope. These results provide theoretical and technical support for condition-based maintenance of composite insulators in power grids.

## 1. Introduction

Composite insulators, composed of glass fiber reinforced epoxy core rods and HTV silicone rubber sheds, have been widely deployed in modern power grids worldwide due to their lightweight, excellent anti-pollution flashover performance, and low maintenance cost [1,2,3]. However, during long-term outdoor service, silicone rubber materials are inevitably subjected to the combined effects of multiple environmental stresses, including UV radiation, temperature-humidity alternation, contamination deposition, and corona discharge. Transport of low-molecular-weight species through silicone rubber depends on the stability of the polymer network and the type and distribution of fillers, which can facilitate the penetration of environmental media and accelerate degradation [4,5,6]. These stresses cause irreversible degradation of silicone rubber, manifested as hydrophobicity loss, surface chalking, cracking, and interfacial failure, which may eventually lead to abnormal heating, flashover, and even catastrophic string breakage accidents [7].

In recent years, extensive research has been conducted on the aging mechanism of silicone rubber insulators, but most studies have focused on the effect of a single aging factor. More broadly, multivariable statistical design methods, including response surface methodology and Taguchi optimization, have been used to identify influential factors in polymer systems and manufacturing processes [8,9]. For example, UV radiation induces chain scission and crosslinking of silicone molecular chains, leading to increased hardness and decreased elasticity [10]; temperature–humidity cycles cause hydrolysis of Si–O–Si backbone bonds and the formation of microcracks [11]; salt and ash contamination form conductive channels on the insulator surface, increasing leakage current and accelerating electrical erosion [12]. Nevertheless, in actual operating environments, insulators are always exposed to the synergistic action of multiple stresses simultaneously, and the aging effect caused by multi-factor synergy is far more severe than the simple superposition of single-factor effects [13,14,15,16,17].

Existing lifetime prediction models mostly rely on single or dual physical parameters such as tensile strength or Shore hardness. Our previous work established a dual-parameter lifetime prediction model based on Shore hardness and Si(−O)_2_ content for silicone rubber insulators in mountainous natural environments [18], but it has three core limitations: (1) it neglects the synergistic effects of multiple environmental stresses and the significant impacts of pollution and electrical factors, limiting its generalization ability in complex operating conditions; (2) it lacks rigorous statistical screening of parameters, leading to potential information redundancy and overfitting problems; and (3) it is validated only with samples from a single manufacturer or environment, with limited engineering generalization ability. Although some studies have attempted to introduce multiple parameters or data-driven algorithms into lifetime prediction [7,10,19], these models often emphasize numerical fitting while providing insufficient physical interpretation of the degradation mechanism and limited independent verification. Therefore, a model that simultaneously considers material degradation, surface pollution, electrical erosion and statistical validation is still needed.

To address the above shortcomings, this study systematically carried out single-factor and multi-factor coupled accelerated aging tests, combined with 45 field samples from the Xuefeng Mountain Natural Aging Station, to reveal the multi-factor synergistic aging mechanism from micro to macro scales. Through rigorous statistical screening, a comprehensive “physical-pollution-electrical” three-dimensional evaluation system was established, and a high-precision multi-parameter synergistic lifetime prediction model was constructed. The real advantage of the proposed model is not only the reduction in prediction error, but also its transparent engineering interpretability: each input variable corresponds to a measurable aging process, including matrix hardening, chemical oxidation, pollution accumulation and electrical erosion. The model was further validated with 15 independent field samples from different manufacturers and operating environments, and its prediction confidence was evaluated using cross-validation and 95% confidence/prediction intervals.

## 2. Experimental Sections

### 2.1. Sample Preparation

The HTV silicone rubber samples used in this study were cut from the sheds of brand-new 110 kV composite insulators produced by manufacturer A, with the same formulation and production process as the field-aged samples. The basic formulation consists of methyl vinyl silicone rubber (90–110 phr) as the matrix, fumed silica (25–40 phr) as the reinforcing filler, and aluminum hydroxide (ATH, 120–130 phr, 5000–6000 meshes) as the flame retardant, along with small amounts of vulcanizing agents and coupling agents, which is consistent with the typical formula widely used in transmission line insulators [18].

All samples were cut into standard test pieces using a sharp blade, with dimensions of 3 × 3 × 2 mm for microstructure and chemical characterization, dumbbell-shaped specimens (75 × 12.5 × 2 mm) for tensile testing, and circular specimens (φ50 × 2 mm) for electrical and hydrophobic property testing. Before testing, all samples were thoroughly wiped with anhydrous ethanol to remove surface floating dust and contaminants and then dried in a vacuum oven at 50 °C for 24 h to eliminate residual moisture.

### 2.2. Accelerated Aging Tests

Accelerated aging tests were conducted in a multi-factor comprehensive aging chamber (Model: Q-Lab QUV/se) that can simultaneously simulate UV radiation, temperature, humidity, and salt spray. All aging stresses were applied simultaneously in the chamber for the comprehensive aging group. Five sets of aging conditions were designed to investigate the individual and combined effects of different environmental stresses:

UV aging: UV irradiation intensity of 0.89 W/m^2^ at 340 nm, with a constant black panel temperature of 50 °C. The test adopts a cyclic mode of 8 h UV irradiation followed by 4 h condensation, with temperature controlled at ±2 °C accuracy. Total duration: 2000 h.

Hygrothermal aging: Constant temperature of 85 °C and relative humidity of 85%, duration of 2000 h.

Salt spray aging: 5 wt% NaCl solution, spray pressure of 0.1 MPa, temperature of 35 °C, duration of 2000 h.

Ash contamination aging: Na_2_SO_4_ ash deposition density of 0.5 mg/cm^2^, temperature of 40 °C, relative humidity of 80%, duration of 2000 h.

Comprehensive aging: Simultaneous application of the above four stresses with the same parameters as individual tests, duration of 2000 h.

The 2000 h comprehensive aging duration was calibrated by performance matching with 10-year field samples from manufacturer A. Shore A hardness, Si(−O)_2_ relative content, contact angle and leakage current were used as benchmark indicators, and the accelerated condition was regarded as equivalent when the comprehensive-aging sample reached the same degradation level as the 10-year field-aged sample. On this basis, the acceleration factor (AF) was calculated as $AF=\frac{t_{field}}{t_{acc}}=\frac{10\;\mathrm{years}\times 365\;\mathrm{days}\times 24\;h\cdot {\mathrm{day}}^{-1}}{2000\;h}=43.8$. Therefore, 1 year of field aging corresponds to approximately 200 h of comprehensive accelerated aging, and the equivalent accelerated-aging durations for 2, 4, 6, 8 and 10 years are 400, 800, 1200, 1600 and 2000 h, respectively. It should be emphasized that this AF is an empirical field-performance-matching factor for the Xuefeng Mountain subtropical mountainous environment rather than a universal Arrhenius-type conversion applicable to all climates. All parameter settings refer to the measured environmental data of typical operating areas in China and the corresponding national standards (GB/T 19893, GB/T 16434 [20,21]).

For each aging condition, 3 parallel samples were set, and each performance test was repeated 3–10 times according to corresponding test standards to ensure data reliability.

### 2.3. Field Samples

Field-aged samples were collected from the Xuefeng Mountain Natural Icing and Aging Station located in Hunan Province, China (latitude 27.5° N, longitude 110.7° E, altitude ~1350 m). This station is characterized by a typical subtropical humid mountain climate with an average annual temperature of 12.6 °C, average annual rainfall of 1800 mm, and annual UV radiation intensity of 180 MJ/m^2^, which represents one of the harshest natural aging environments for composite insulators in China.

A total of 45 composite insulator samples with service years of 0, 2, 4, 6, 8, and 10 years were collected, all from manufacturer A and operating under the same environmental conditions. All samples were cut from the middle part of the middle insulator sheds, within 5 cm around the core rod to ensure consistency. Before testing, all samples were conditioned in a constant temperature and humidity chamber (23 °C, 50% RH) for 24 h to eliminate the interference of environmental variables.

Referring to the Chinese power industry standard DL/T 627 [22], the failure criterion (end of service life) of composite insulators is defined as: static contact angle drops below 60° (complete loss of hydrophobicity), severe surface chalking, and tensile strength retention rate lower than 60%. The 10-year field samples from the station are close to this failure threshold, thus 10 years is set as the reference total life (T_(F/A)_ = 10 years) of insulators under this environmental condition.

In addition, 15 independent samples from three different manufacturers (represented by manufacturer B) operating in farmland, coastal, industrial, and mixed environments were collected for model validation. The formulation difference between manufacturers is consistent with the settings in the reference [18].

### 2.4. Characterization and Performance Testing

(1) Microstructure characterization. Surface microstructure changes in the silicone rubber samples were observed using a field-emission scanning electron microscope (FE-SEM; Regulus 8100, Hitachi High-Tech Corporation, Tokyo, Japan) at an accelerating voltage of 5 kV. Before observation, all samples were sputter-coated with a gold layer approximately 10 nm thick to improve electrical conductivity.

(2) Chemical-structure analysis. Fourier-transform infrared spectroscopy (FTIR) measurements were performed using a Nicolet iS10 spectrometer (Thermo Fisher Scientific, Waltham, MA, USA) in attenuated-total-reflection mode. The wavenumber range was 4000–400 cm^−1^, with a resolution of 4 cm^−1^ and 32 scans per sample. The relative content of each functional group was calculated by normalizing its peak area to that of the Si–O–Si peak at 1010 cm^−1^.

X-ray photoelectron spectroscopy (XPS) analysis was carried out using a Thermo Scientific K-Alpha spectrometer (Thermo Fisher Scientific) with a monochromatic Al Kα X-ray source (1486.6 eV). The binding energy was calibrated using the contaminated carbon C1s peak at 284.80 eV. The Si2p peak was deconvoluted into three components corresponding to Si(−O)_2_ (101.2 eV), Si(−O)_3_ (102.1 eV), and Si(−O)_4_ (102.8 eV) using the XPS Peak 4.1 software.

Physical Property Testing: Shore A hardness was measured using a Shore A durometer (Model: LX-A, Shanghai Liuling Instrument Co., Ltd., Shanghai, China) according to the ISO 868 standard [23]. Six test points were evenly distributed on each sample, and the average value was taken. Tensile strength and elongation at break were tested using an electronic universal testing machine (Model: TSKL-500 N, Tinius Kuli, Suzhou, China) at a stretching rate of 500 mm/min according to the ISO 37 standard [24]. Three parallel samples were tested for each condition, and the average value was reported.

Electrical and Contamination Property Testing: Volume resistivity was measured using a high-resistance meter (Model: Keithley 6517B, Keithley Instruments, Solon, OH, USA) at 1000 V DC according to the GB/T 1410 standard [25]. The test was performed in a constant temperature and humidity chamber (23 °C, 50% RH) after the samples were conditioned for 24 h. Static water contact angle was measured using a contact angle analyzer (Model: KRÜSS DSA25, KRÜSS GmbH, Hamburg, Germany) according to the IEC/TS 62073 standard [26]. A 5 μL deionized water droplet was deposited on the sample surface, and the contact angle was measured immediately. Ten measurements were taken for each sample, and the average value was calculated. Equivalent salt deposit density (ESDD) and non-soluble deposit density (NSDD) were quantitatively tested according to the DL/T 864 standard [27]. Leakage current under wet conditions was measured using a precision current meter (Model: Keithley 6485, Keithley Instruments, Solon, OH, USA) at an applied voltage of 10 kV AC. Electrical erosion depth was measured using a confocal laser scanning microscope (Model: Olympus LEXT OLS4000, Olympus Corporation, Tokyo, Japan).

## 3. Results and Discussion

### 3.1. Aging Performance Evolution and Microscopic Mechanism

**Figure 1:**
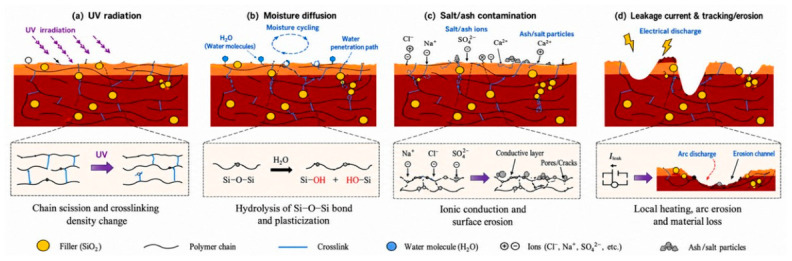
Schematic illustration of the main aging mechanisms of HTV silicone rubber composite insulators: (**a**) UV radiation induces chain scission and crosslinking density change; (**b**) moisture diffusion causes hydrolysis of the Si–O–Si bond and plasticization; (**c**) salt/ash contamination leads to ionic conduction and surface erosion; (**d**) leakage current results in local heating, arc erosion and material loss.

The main aging mechanisms of HTV silicone rubber under different environmental stresses are schematically illustrated in Figure 1. UV radiation (Figure 1a) with high photon energy can directly break the Si-C bonds in the silicone rubber molecular chains, generating free radicals such as ·H and ·CH_3_. These free radicals can either recombine to form new cross-linked bonds or react with oxygen in the air to form polar groups such as hydroxyl (–OH) and carbonyl (C=O), leading to changes in crosslink density and loss of hydrophobicity.

Moisture diffusion (Figure 1b) induces hydrolysis of the Si–O–Si backbone bonds, breaking the long molecular chains into shorter segments and reducing the mechanical strength of the material. In addition, moisture can plasticize the silicone rubber matrix, further decreasing its hardness and elasticity. Salt and ash contamination (Figure 1c) deposit on the insulator surface and form a conductive layer when wetted by rain or fog, leading to ionic conduction and increased leakage current. The leakage current generates local Joule heating (Figure 1d) and arc discharge, causing material erosion, tracking, and even carbonization, which further accelerates the degradation process.

The synergistic effect of these four factors creates a positive feedback loop that significantly accelerates the aging rate of HTV silicone rubber. For example, UV-induced chain scission creates more defects and active sites, which are more susceptible to hydrolysis and chemical attack. Moisture ingress enhances the conductivity of contamination layers, increasing leakage current and local heating, which in turn accelerates thermal degradation and evaporation of low-molecular-weight siloxanes. This synergistic effect explains why the actual service life of insulators in complex environments is often much shorter than predicted by single-factor aging tests.

Compared with previous multifactor aging studies on HTV silicone rubber, this work couples four environmental stresses (UV radiation, humidity, salt, and ash con-tamination) and verifies the resulting aging trend using field samples covering the fully investigated service period. This design more closely represents the combined degrada-tion processes experienced by insulators in complex operating environments.

#### 3.1.1. Surface Microstructure Evolution

**Figure 2:**
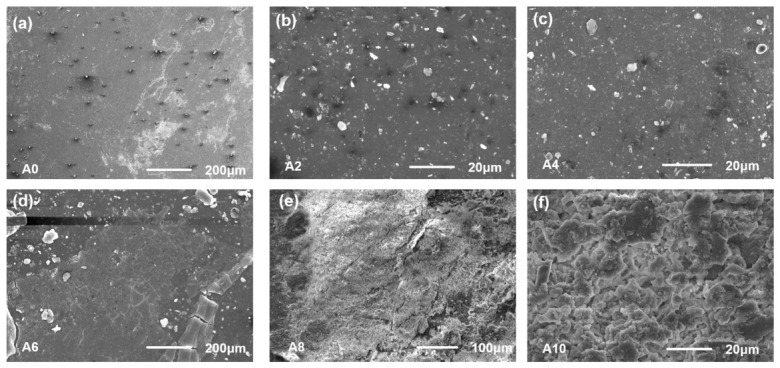
SEM micrographs of HTV silicone rubber samples with different operating years: (**a**) A0 (0 year), (**b**) A2 (2 years), (**c**) A4 (4 years), (**d**) A6 (6 years), (**e**) A8 (8 years), (**f**) A10 (10 years).

Surface morphology evolution observed by SEM further reveals the progressive microstructural degradation process of HTV silicone rubber during long-term field operation, as shown in Figure 2. The unaged sample (A0, Figure 2a) exhibits a smooth and dense surface with only a few scattered tiny pores, and the fumed silica filler particles are uniformly embedded in the silicone rubber matrix without any exposure or agglomeration. After 2 years of operation (A2, Figure 2b), the surface becomes slightly rough, and isolated white filler particles and small dark micropores begin to appear, which are attributed to the initial UV-induced chain scission and slight hydrolysis of the Si–O–Si backbone. For the 4-year-aged sample (A4, Figure 2c), the number and size of exposed filler particles increase significantly, and the surface roughness further increases, indicating the gradual degradation of the polymer matrix surrounding the fillers.

After 6 years of service (A6, Figure 2d), obvious linear microcracks and large-sized degradation products appear on the surface, and the microcracks start to propagate along the filler–matrix interface due to the accumulated internal stress caused by crosslinking and hydrolysis reactions. For the 8-year-aged sample (A8, Figure 2e), microcracks coalesce to form interconnected crack networks, and large areas of the polymer matrix are eroded, leading to severe surface roughening and partial chalking. After 10 years of operation (A10, Figure 2f), the surface shows complete and severe chalking with a typical granular and porous structure, where almost all the silicone rubber matrix is degraded and the silica fillers are fully exposed and agglomerated. This indicates that the silicone rubber has entered the final accelerated aging stage with drastically deteriorated mechanical and electrical properties.

In addition, the comprehensively aged sample exhibits more severe surface degradation than any single-factor aged sample, confirming that the synergistic effect of UV radiation, humidity, salt contamination and electrical stress markedly accelerates the microstructural damage of HTV silicone rubber.

Semi-quantitative analysis was performed using ImageJ software (version 1.54g; National Institutes of Health, Bethesda, MD, USA). For each sample, five randomly selected fields of view at the same magnification were processed with a unified binarization threshold determined from the grayscale histogram, and the exposed filler area fraction and average crack width were calculated. The exposed-filler area fraction rises from 2.1 ± 0.5% for the unaged sample to 41.8 ± 3.6% for the 10-year-aged sample, and the average crack width increases from nearly undetectable at 4 years to 2.8 ± 0.4 μm at 10 years. These microstructural variations are highly consistent with the three-stage aging behavior observed in macroscopic performance tests, indicating that filler exposure and crack network coalescence are the core microscopic drivers for the transition from the stable aging stage to the accelerated failure stage.

#### 3.1.2. Chemical Structure Evolution

**Figure 3:**
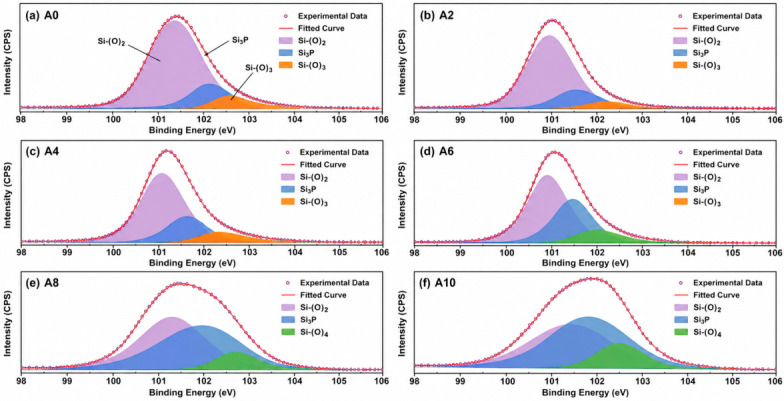
XPS Si2p peak fitting spectra of HTV silicone rubber samples with different operating years: (**a**) A0 (0 year), (**b**) A2 (2 years), (**c**) A4 (4 years), (**d**) A6 (6 years), (**e**) A8 (8 years), (**f**) A10 (10 years). The peaks are deconvoluted into Si(−O)_2_ (purple), Si_2_p (blue), Si(−O)_3_ (orange) and Si(−O)_4_ (green) components.

XPS Si2p peak fitting results (Figure 3) show that the Si2p peak of the unaged sample is mainly composed of Si(−O)_2_ (101.2 eV) and a small amount of Si(−O)_3_ (102.1 eV). With the increase in aging time, the relative content of Si(−O)_2_ gradually decreases, while the relative contents of Si(−O)_3_ and Si(−O)_4_ (102.8 eV) gradually increase [18]. This is because the Si–O–Si main chain breaks under environmental stresses, and the broken bonds combine oxygen and water molecules to form higher-coordinated Si-O structures, which are consistent with the FTIR analysis results.

**Figure 4:**
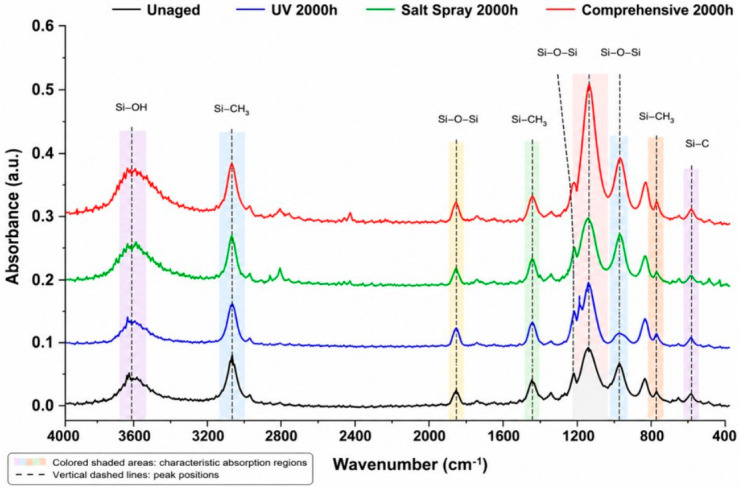
FTIR spectra of HTV silicone rubber samples under different aging conditions: unaged (black), UV 2000 h (blue), salt spray 2000 h (green), and comprehensive aging 2000 h (red). The characteristic peaks of key functional groups are marked.

FTIR spectra (Figure 4) show that the relative peak areas of Si–(CH_3_)_2_ side groups, CH_3_ in Si-CH_3_ (1260 cm^−1^) and Si–O–Si main chains (1010 cm^−1^) all decrease significantly after aging [18,28], while the intensity of the –OH peak (3580 cm^−1^) shows a trend of first increasing and then decreasing [18,28,29,30,31]. This indicates that the Si-C bonds of side methyl groups are broken and the Si–O–Si main chains are hydrolyzed, generating polar hydroxyl groups and resulting in the loss of material hydrophobicity. The subsequent decrease in –OH content is attributed to the decomposition of ATH filler, which consumes many hydroxyl groups to form Al_2_O_3_ crystals, which is consistent with the precipitate phenomenon observed in SEM images. The characteristic peak changes in the comprehensively aged sample are the most significant, further verifying the promoting effect of multi-factor synergy on chemical degradation.

To realize quantitative analysis of FTIR spectra, the peak area of Si–O–Si at 1010 cm^−1^ was taken as the internal reference, and the relative peak area change rates of Si–(CH_3_)_2_ and –OH under different aging conditions and service years were calculated. The results show that after 2000 h of comprehensive aging, the relative content of Si–(CH_3_)_2_ decreases by 68%, while the relative content of –OH increases by 52% in the early stage and then decreases by 21% in the later stage, which is consistent with the three-stage aging law.

#### 3.1.3. Macroscopic Performance Evolution

**Figure 5:**
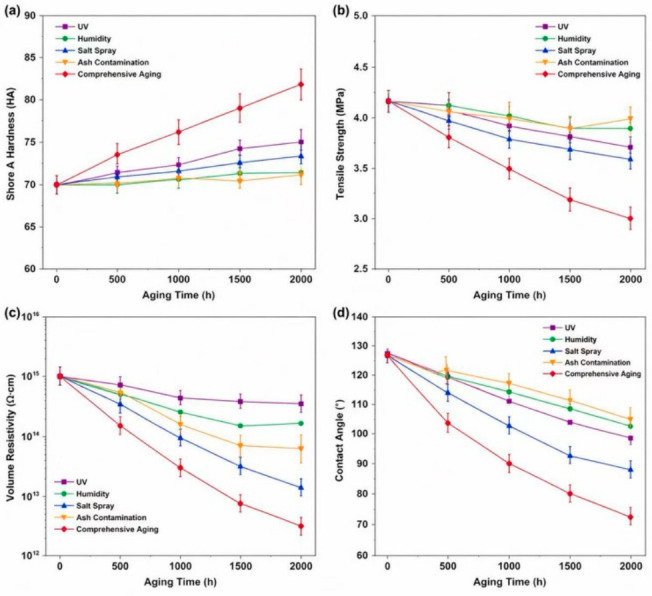
Variation in key performance indicators of HTV silicone rubber under different accelerated aging conditions: (**a**) Shore A hardness, (**b**) tensile strength, (**c**) volume resistivity, (**d**) contact angle. The error bars represent the standard deviation of three parallel measurements.

The variation in key performance indicators of HTV silicone rubber under different accelerated aging conditions is shown in Figure 5, with standard deviation error bars for all quantitative results. The Shore A hardness of all samples increases with aging time (Figure 5a), with the comprehensively aged sample showing the largest increase (from 70 HA to 82 HA after 2000 h). The increase in hardness is mainly attributed to the crosslinking reaction of silicone rubber molecular chains caused by aging. UV radiation has the most significant effect on hardness increase because high-energy photons can directly break the Si-C bonds, generating free radicals that induce crosslinking.

The tensile strength of all samples decreases with aging time (Figure 5b), with the comprehensively aged sample retaining only 72% of its initial strength. The decrease in tensile strength is caused by the breakage of molecular main chains and the degradation of the filler–matrix interface. Salt spray aging has a significant effect on tensile strength because salt ions can penetrate the material, causing interfacial debonding between the filler and the silicone rubber matrix.

The volume resistivity of all samples decreases exponentially with aging time (Figure 5c), with the comprehensively aged sample showing a three-order-of-magnitude decrease. The decrease in volume resistivity is mainly due to the formation of conductive channels in the material. Salt and ash contamination have the most significant effect on volume resistivity because soluble salts and conductive ash particles form continuous conductive paths on the surface and inside the material.

The contact angle of all samples decreases with aging time (Figure 5d), indicating the loss of hydrophobicity. The contact angle of the comprehensively aged sample is only 72°, which is close to the hydrophilic threshold (60°), indicating a high risk of pollution flashover. The loss of hydrophobicity is caused by the oxidation of methyl groups to polar hydroxyl groups and the increase in surface roughness.

Overall, the aging process of HTV silicone rubber presents a three-stage nonlinear progression: plateau stage (0–4 years), rising stage (4–8 years), and acceleration stage (8–10 years), which is consistent with the evolution law of microstructure and chemical structure.

### 3.2. Key Parameter Screening and Statistical Analysis

To avoid information redundancy and overfitting, Pearson correlation analysis and *t*-tests were performed on 12 potential aging parameters to screen key parameters that are significantly correlated with service time and independent of each other [32,33].

The Pearson correlation coefficient (r) was first calculated to quantify the linear correlation between each aging parameter and the actual service time of insulators, with the formula defined as [32,33]

(1) $$\begin{array}{c} \begin{array}{c} \begin{array}{c} r=\frac{n\sum xy-\sum x\sum y}{\sqrt{\left(n\sum x^{2}-{\left(\sum x\right)}^{2})(n\sum y^{2}-{\left(\sum y\right)}^{2}\right)}} \end{array} \end{array} \end{array}$$

where x is the measured value of a candidate aging parameter, y is the actual service time of the corresponding insulator sample, and n is the total number of training samples (n=45 in this study).

To verify the statistical significance of the calculated correlation coefficient, a two-tailed t-test was conducted. The t-value was calculated using the following formula [34]:

(2) $$\begin{array}{c} \begin{array}{c} t=\frac{r\sqrt{n-2}}{\sqrt{1-r^{2}}} \end{array} \end{array}$$

where r is the Pearson correlation coefficient, and the degree of freedom is n−2. Based on the obtained t-value and degree of freedom, the *p*-value was calculated to evaluate the significance level [34]:

(3) $$\begin{array}{c} p=2\cdot P(T>|t|,df=n-2) \end{array}$$

where P(⋅) represents the cumulative distribution function of the t-distribution. In this study, a parameter was considered to have a statistically significant correlation with service time when p<0.05, and parameters with p>0.05 were excluded from the subsequent model construction. Parameters with p<0.005 and no significant collinearity between each other were selected as the core input variables of the model to ensure the robustness and interpretability of the regression results.

The calculation results of the correlation coefficient, t-value, *p*-value, and significance judgment for all candidate parameters are shown in Table 1.

Parameters including tensile strength, contact angle and volume resistivity were excluded from the model for two reasons: (1) their correlation with service time is lower than the selected core parameters, with weaker statistical significance; (2) they have strong collinearity with the selected parameters, which would introduce information redundancy and overfitting risk. The variance inflation factor (VIF) test confirms that all selected parameters have VIF values less than 5, indicating no severe multicollinearity.

Six parameters with *p* < 0.005 and no significant collinearity with each other were finally selected as key aging parameters: Shore A hardness (A), Si(−O)_2_ relative content (X_SiO_), ESDD, NSDD, leakage current (I), and erosion depth (T_E_). These parameters cover the physical, chemical, pollution, and electrical aspects of insulator aging, forming a comprehensive “physical-pollution-electrical” three-dimensional evaluation system.

The six key aging parameters obtained through screening exhibit significant differences in dimensions and orders of magnitude (e.g., Shore hardness is in HA with a value range of 65–86; leakage current is in mA with a value range of 320–820). If linear regression is performed directly, parameters with larger values will exert a dominant influence on the model, resulting in regression coefficients that cannot truly reflect the contribution weight of each indicator to aging. Therefore, the Z-score standardization method is first applied to preprocess all parameters to eliminate dimensional differences:

(4) $$Z_{x}=\frac{X_{i}-\bar{x}}{\sigma_{x}}$$

where $Z_{x}$ is the standardized value of parameter x, $\bar{x}$ is the mean value of parameter x in the 45 training samples, and $\sigma_{x}$ is the standard deviation of parameter x. The mean and standard deviation of each key parameter obtained based on the training sample statistics are shown in Table 2.

### 3.3. Lifetime Prediction Model Construction and Validation

Taking the standardized equivalent operating time $Z_{T_{C/A}}$ as the dependent variable and the six standardized key parameters as the independent variables, a prediction model is constructed using the multiple stepwise regression method. The stepwise regression method automatically screens variables that contribute significantly to the dependent variable and eliminates redundant information by setting a variable entry threshold (p<0.05) and a rejection threshold (p>0.1), effectively avoiding the overfitting problem. The fitting process adopts the least squares method with the criterion of minimizing the sum of squared residuals ∑i=1n(TC/A,i−T^C/A,i)2, where $T_{C/A,i}$ is the actual equivalent operating time of the i-th sample and T^C/A,i is the predicted value of the model.

The order in which variables enter the model step by step is Shore hardness (p<0.001) → relative content of $Si(-O{)}_{2}$ (p<0.001) → leakage current (p<0.001) → erosion depth (p<0.001) → equivalent salt deposit density (p=0.002) → non-soluble deposit density (p=0.003). All six parameters meet the entry threshold and no variables are eliminated, indicating that the screened key parameters all have independent and significant contributions to insulator aging. The final standardized multiple linear regression model is obtained:

(5) $$Z_{T_{C/A}}=0.12Z_{A}-11.85Z_{X_{SiO}}+0.08Z_{ESDD}+0.05Z_{NSDD}+0.03Z_{I}+0.06Z_{T_{E}}+0.92$$

Note: Equation (5) presents dimensionless standardized regression coefficients after Z-score normalization, which reflects the relative contribution weight of each parameter.

Compared with existing single-parameter or dual-parameter lifetime models, the proposed model has three practical advantages. First, the selected variables are physically interpretable and cover different degradation pathways, rather than only describing one macroscopic property. Second, Pearson correlation, significance testing and VIF diagnosis are used before regression, which reduces redundant inputs and improves model stability. Third, the model is evaluated using both internal cross-validation and external independent field samples, making the reported accuracy more representative of engineering applications than a single training-set fitting result. These features explain why the proposed model performs better than the traditional dual-parameter model while retaining clear physical meaning.

The coefficient of determination $R^{2}$ of the model is 0.92, and the adjusted coefficient of determination $R_{adj}^{2}$ is 0.91, indicating that the model can explain 91% of the variation in equivalent operating time with a good fitting effect. $R^{2}$ is calculated as the ratio of the sum of squares of regression to the total sum of squares and adjusted $R^{2}$ corrects $R^{2}$ according to the number of independent variables and sample size to avoid overestimation of fitting accuracy caused by multiple variables.

Specifically, the goodness-of-fit statistics were calculated as $R^{2}=1-\frac{SS_{res}}{SS_{tot}}$ and $\;R_{adj}^{2}=1-\frac{\left(1-R^{2})(n-1\right)}{n-k-1}$, where $SS_{res}$ is the residual sum of squares, $SS_{tot}\;$is the total sum of squares, n is the sample size, and k is the number of independent variables.

**Figure 6:**
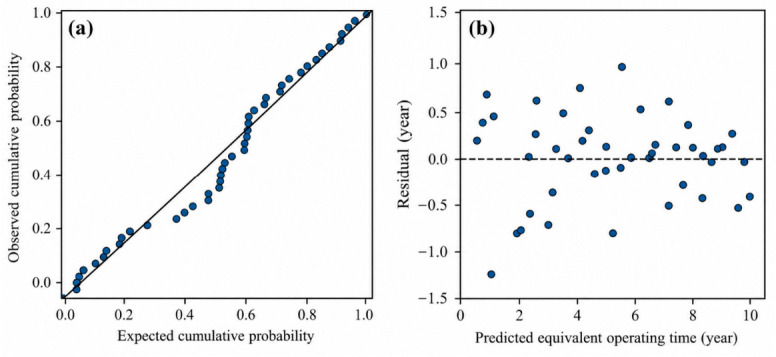
Residual analysis plots of the lifetime prediction model: (**a**) normal probability P-P plot of residuals; (**b**) residual scatter plot for the homoscedasticity test.

Residual analysis was performed to verify the validity of the linear regression. As shown in Figure 6, the residuals follow an approximately normal distribution with a mean close to 0, and no obvious heteroscedasticity is observed in the residual scatter plot, satisfying the basic assumptions of linear regression.

Since the coefficients of the standardized model only reflect the relative contribution of each parameter and cannot be directly used for practical engineering prediction, it is necessary to restore it to a prediction model based on original physical quantities. According to the inverse transformation of Z-score standardization, the restoration formula for equivalent operating time is

(6) $$T_{C/A}=Z_{T_{C/A}}\times \sigma_{T_{C/A}}+{\bar{T}}_{C/A}$$

where ${\bar{T}}_{C/A}=5.0$ years is the mean value of the equivalent operating time of the training samples, and $\sigma_{T_{C/A}}=3.2$ years is its standard deviation. Substituting Equation (5) into Equation (6) and performing coefficient conversion combined with the mean and standard deviation of each parameter in Table 2, the final prediction formula for equivalent operating time based on original physical quantities is obtained:

(7) $$T_{C/A}=0.12A-11.85X_{SiO}+0.08ESDD+0.05NSDD+0.03I+0.06T_{E}+0.92$$

Note: Equation (7) presents regression coefficients with actual physical units, which can be directly used for engineering calculation. The numerical coincidence with Equation (5) is caused by the dimensional transformation in this study, and their physical meanings are completely different.

The actual values, physical units, standard errors, *p*-values and 95% confidence intervals of each coefficient in Equation (7) are listed in Table 3.

#### 3.3.1. Concept of Equivalent Operating Time and Linear Proportional Hypothesis

Equivalent operating time is defined as a unified metric of aging degree, which converts the aging degree of insulators under different environments and from different manufacturers into the corresponding operating duration under the standard reference environment (Xuefeng Mountain Natural Aging Station). For samples from the same manufacturer and the same environment, the equivalent operating time is consistent with the actual service time; for samples from different environments/manufacturers, the equivalent operating time reflects the relative aging degree, and the actual remaining life can be calculated through the proportional relationship.

This study is based on the basic assumption that the aging degree of insulators is proportional to the equivalent operating time, i.e., the proportion of consumed life is linearly correlated with the material degradation level. The rationality of this hypothesis is supported by three aspects:

Experimental data: Within the 0–10 year service cycle, the core aging parameters (Shore A hardness, Si(−O)_2_ content) show a significant linear correlation with service time ($R^{2}>0.79$), satisfying the premise of linear relationship;

Literature support: Numerous studies have confirmed that in the early and middle aging stages of silicone rubber insulators, the material degradation rate is relatively stable, and the aging degree is approximately linearly proportional to service time [18];

Validation: The field sample verification shows that the prediction error under this hypothesis is within the acceptable range of engineering applications.

For insulators manufactured by different vendors, although their absolute total service life may vary due to differences in raw material formulation and manufacturing process, the proportional relationship between consumed life and aging degree remains consistent under the same environmental stress. Specifically, the ratio of the actual operating time $T_{C}$ to the total life $T_{F}$ of the target insulator should be identical to the ratio of the equivalent operating time $T_{C/A}$ to the reference total life $T_{F/A}$ of the standard reference insulator (from the Xuefeng Mountain Natural Aging Station), which can be mathematically expressed as

(8) $$\frac{T_{C}}{T_{F}}=\frac{T_{C/A}}{T_{F/A}}$$

where $T_{C}$ denotes the actual on-site operating time of the target insulator, $T_{F}$ denotes the total service life of the target insulator, $T_{C/A}$ denotes the equivalent operating time of the target insulator calculated by the proposed multi-parameter model, and $T_{F/A}$ denotes the reference total life of HTV silicone rubber insulators, which is determined as 10 years based on the full life cycle samples (0–10 years) collected from the Xuefeng Mountain Aging Station.

The remaining service life $T_{R}$ of the insulator is defined as the difference between its total service life and the actual operating time that has been consumed:

(9) $$T_{R}=T_{F}-T_{C}$$

Substituting the proportional relationship in Equation (8) into Equation (9), we can derive the general remaining life prediction formula applicable to insulators from different manufacturers:

(10) $$T_{R}=\frac{T_{C}}{T_{C/A}}\times T_{F/A}-T_{C}$$

Note: Both sides of Equation (10) have the dimension of time (year), with complete dimensional consistency. The formula is based on the relative proportional relationship between aging degree and consumed life and is applicable to insulators from different manufacturers.

Substituting $T_{F/A}$ = 10 years and Equation (7) (the equivalent operating time prediction formula based on original physical quantities) into the above equation, the final remaining life prediction formula is obtained:

(11) $$T_{R}=\frac{T_{C}}{0.12A-11.85X_{SiO}+0.08ESDD+0.05NSDD+0.03I+0.06T_{E}+0.92}\cdot 10-T_{C}$$

#### 3.3.2. Model Validation and Error Analysis

To verify the accuracy, robustness and generalization ability of the proposed model, the validation strategy was expanded from a single error comparison to three levels: (i) same-manufacturer hold-out testing, (ii) cross-manufacturer and cross-environment independent testing, and (iii) 5-fold cross-validation of the 45 training samples. The independent validation set contains 15 field-aged insulator samples from farmland, coastal, industrial and mixed environments, which were not used for coefficient fitting. This design allows the model to be evaluated under both interpolation and extrapolation conditions.

The detailed information of the 15 independent validation samples is shown in Table 4.

The per-sample prediction results of the 15 independent validation samples are summarized in Table 5, including the predicted equivalent operating time, absolute error and relative error for each sample. For same-manufacturer samples, all absolute prediction errors are within 1.0 years, and the relative error decreases gradually with the extension of service time. For cross-manufacturer samples covering coastal, industrial and mixed environments, most samples have errors within 1.5 years, and the overall error level remains within the acceptable range for engineering condition assessment.

To make independent testing more transparent, Table 6 summarizes the validation metrics, confidence intervals and prediction-interval coverage for the proposed model and the traditional dual-parameter model.

For each validation sample, the prediction error $\epsilon_{i}$ is defined as [19]

(12) ϵi=y^i−yi

where y^i is the predicted equivalent operating time of the i-th sample, and $y_{i}$ is the actual service time of the i-th sample. The mean absolute error (MAE) of all validation samples was calculated to quantitatively evaluate the model’s prediction performance.

The validation results show that the average prediction error (MAE) of the proposed model is 0.55 years for same-manufacturer samples (No. 1–5), with a 95% confidence interval of 0.39–0.71 years. For cross-manufacturer samples (No. 6–15), the MAE is 1.0 year, with a 95% confidence interval of 0.74–1.26 years. The overall independent test MAE is 0.85 years and 14 of 15 validation samples fall within the 95% prediction interval, indicating acceptable uncertainty control. In contrast, the traditional dual-parameter model gives an average error of approximately 1.5 years, so the proposed model improves the prediction accuracy by about 33%.

**Figure 7:**
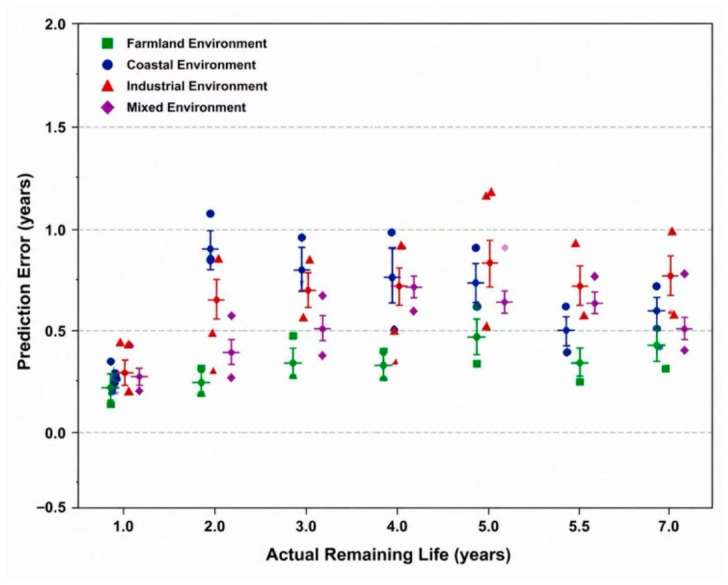
Distribution of absolute prediction errors for the proposed multi-parameter lifetime model across farmland, coastal, industrial, and mixed operating environments. The plotted values correspond to the 15 independent validation samples listed in Table 5.

The prediction error distribution under different actual remaining lifetimes and operating environments is shown in Figure 7. Coastal and industrial samples show slightly larger error dispersion than farmland samples because surface contamination and leakage-current fluctuation are more pronounced in these environments; nevertheless, the absolute errors remain within the acceptable range for maintenance decision-making.

**Figure 8:**
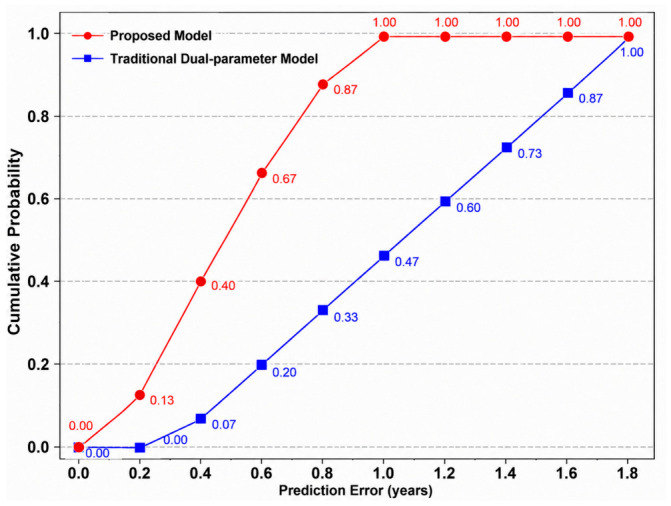
Cumulative probability distribution of prediction errors for the proposed multi-parameter model and the traditional dual-parameter model. The numbers on the curves indicate the cumulative probability values at corresponding error points.

The cumulative probability distribution of prediction errors compared with the traditional dual-parameter model is shown in Figure 8. The proposed model shifts the cumulative error curve toward the low-error region, demonstrating that the added pollution and electrical-erosion indicators provide effective information beyond Shore hardness and Si(−O)_2_ content alone.

To further verify the model’s robustness and avoid overfitting, 5-fold cross-validation was performed on the 45 training samples. The samples were randomly divided into five subsets with approximately nine samples in each fold; in each round, four folds were used for model training, and the remaining fold was used for testing. The process was repeated until each fold was used once as the testing set, and the complete procedure was repeated ten times with different random partitions. The average cross-validation MAE is 0.62 ± 0.08 years, RMSE is 0.78 ± 0.11 years, and validation R^2^ is 0.89 ± 0.03, which are close to the independent-test results and demonstrate that the model does not rely on a particular sample split.

#### 3.3.3. Comparison with Similar Studies and Model Applicability

Compared with single-factor aging studies that mainly explain UV, humidity or pollution effects separately [11,12,13,14,15,16,17], this work combines accelerated aging, field verification and multi-source parameters to describe the coupled degradation process. The SEM, XPS and FTIR results jointly show that surface cracking, filler exposure, Si(−O)_2_ consumption and hydrophobicity loss occur in a correlated manner, which supports the use of a multi-parameter prediction framework rather than a single indicator.

Compared with the dual-parameter model based on Shore hardness and Si(−O)_2_ content [18], the present model adds ESDD, NSDD, leakage current and erosion depth, thereby incorporating pollution accumulation and electrical erosion into the lifetime calculation. This addition is the main reason for the improved cross-manufacturer prediction accuracy. Compared with purely data-driven prediction methods [7,10,19], the present regression model is less complex but more transparent because the sign and magnitude of each coefficient can be directly linked to a specific aging mechanism. Therefore, the proposed model is more suitable for engineering condition assessment when the number of field samples is limited and interpretability is required.

#### 3.3.4. Limitations and Future Work

This study has made important progress in revealing the multi-factor aging mechanism and establishing a high-precision lifetime prediction model for HTV silicone rubber composite insulators, but there are still some limitations that need to be addressed in future work:

Sample coverage limitation: The field samples used in this study are mainly from central China with a humid subtropical climate. The aging characteristics of insulators in extreme environments such as high altitude, severe cold, and strong UV radiation may be different. Future work will expand the sample collection to cover more diverse climatic regions to further improve the model’s generalization ability.

Service life range limitation: The current model is validated for insulators with a service life of 0–10 years. The aging mechanism and performance evolution law of insulators with a service life exceeding 10 years need to be further studied, and the model should be extended to cover longer service periods.

Nonlinear coupling quantification: The current model is a statistical regression model that does not fully quantify the nonlinear coupling effects between different aging factors. Future work will combine molecular dynamics simulation and finite element analysis to establish a more physically based aging model that can accurately describe the synergistic effects of multiple factors.

Online monitoring integration: The current model requires laboratory testing of parameters, which is time-consuming and labor-intensive. Future work will develop miniaturized online monitoring sensors for key aging parameters and integrate the lifetime prediction model with the online monitoring system to realize real-time evaluation and early warning of insulator aging status.

## 4. Conclusions

In this study, the multi-factor aging mechanism of HTV silicone rubber composite insulators was systematically investigated, and a high-precision multi-parameter synergistic lifetime prediction model was established. The main conclusions are as follows:

The aging of HTV silicone rubber under different environmental stresses follows a three-stage nonlinear progression: plateau stage (0–4 years), rising stage (4–8 years), and acceleration stage (8–10 years). The combined effect of UV radiation, humidity, salt contamination, and ash contamination is significantly more severe than any single factor, leading to faster degradation of material properties.

The four key performance indicators (Shore A hardness, tensile strength, volume resistivity, and contact angle) all show regular changes with aging time. Shore A hardness increases, while tensile strength, volume resistivity, and contact angle decrease. Among the single factors, UV radiation has the most significant effect on hardness and tensile strength, while salt contamination has the most significant effect on volume resistivity and contact angle.

A “physical-pollution-electrical” three-dimensional evaluation system was established by selecting six key aging parameters (Shore A hardness, Si(−O)_2_ relative content, ESDD, NSDD, leakage current, and erosion depth) through rigorous statistical screening. The acceleration factor of the comprehensive accelerated-aging test was calculated as 43.8 by matching 2000 h accelerated aging to 10-year field degradation. The constructed multi-parameter synergistic lifetime prediction model achieves an average prediction error of 1.0 years for cross-manufacturer samples, with the cross-validation MAE of 0.62 ± 0.08 years and most independent validation samples falling within the 95% prediction intervals. Compared with traditional dual-parameter models, the proposed model improves prediction accuracy by about 33% and provides clearer physical interpretability.

This study provides a comprehensive understanding of the multi-factor aging mechanism of silicone rubber insulators and a reliable technical method for lifetime prediction. The proposed model can effectively guide condition-based maintenance and replacement strategies of composite insulators in power grids within the present validation scope. Future work will further expand the field-sample database across more climatic regions and incorporate nonlinear coupling models to improve generalization under extreme environments.

## Figures and Tables

**Table 1:** Statistical results of correlation among various parameters.

Parameter	Correlation Coefficient r	t-Value	*p* -Value	Significance	VIF
Shore A hardness	0.82	6.71	<0.001	Extremely significant	1.82
Si(−O)_2_ relative content	−0.79	−5.98	<0.001	Extremely significant	2.31
ESDD	0.65	4.32	0.002	Highly significant	1.64
NSDD	0.61	3.95	0.003	Highly significant	1.76
Leakage current	0.75	5.23	<0.001	Extremely significant	2.15
Erosion depth	0.73	4.98	<0.001	Extremely significant	1.93
Tensile strength	−0.58	3.62	0.007	Significant	3.84
Elongation at break	−0.52	3.15	0.015	Significant	3.27
Contact angle	−0.48	2.87	0.023	Significant	4.18
Volume resistivity	−0.45	2.64	0.031	Significant	4.02
Tear strength	−0.42	2.41	0.042	Significant	3.15
Si-CH_3_ relative content	−0.38	2.16	0.065	Not significant	–

**Table 2:** Statistical characteristics of key aging parameters (training samples, n = 45).

Parameter	Mean $\bar{x}$	Standard Deviation $\sigma_{x}$	Unit
Shore hardness A	74.8	5.2	HA
Relative content of $Si{\left(-O\right)}_{2}$ $X_{SiO}$	0.58	0.09	−
Equivalent salt deposit density ESDD	0.14	0.05	mg/cm^2^
Non-soluble deposit density NSDD	0.87	0.16	mg/cm^2^
Leakage current I	560	145	mA
Erosion depth $T_{E}$	1.9	0.7	mm
Equivalent operating time $T_{C/A}$	5.0	3.2	year

**Table 3:** Regression coefficients of the lifetime prediction model with physical units.

Parameter	Coefficient Value	Unit	Standard Error	*p*-Value	95% Confidence Interval
Shore hardness A	0.12	year·HA^−1^	0.018	<0.001	[0.084, 0.156]
Si(−O)_2_ relative content XSiO	−11.85	year	1.24	<0.001	[−14.33, −9.37]
ESDD	0.08	year·cm^2^·mg^−1^	0.021	0.002	[0.038, 0.122]
NSDD	0.05	year·cm^2^·mg^−1^	0.015	0.003	[0.020, 0.080]
Leakage current I	0.03	year·mA^−1^	0.007	<0.001	[0.016, 0.044]
Erosion depth TE	0.06	year·mm^−1^	0.013	<0.001	[0.034, 0.086]
Constant term	0.92	year	0.31	0.004	[0.30, 1.54]

**Table 4:** Detailed information of 15 independent validation samples.

Sample No.	Manufacturer	Environment Type	Actual Service Time (Year)	Shore A Hardness (HA)	Si(−O)_2_ Relative Content	ESDD (mg/cm^2^)	NSDD (mg/cm^2^)	Leakage Current (mA)	Erosion Depth (mm)
1	A	Farmland	2.0	68.8	0.72	0.08	0.62	380	1.1
2	A	Farmland	4.0	72.2	0.53	0.11	0.78	490	1.6
3	A	Farmland	6.0	73.0	0.36	0.15	0.91	610	2.0
4	A	Farmland	8.0	75.7	0.30	0.18	1.02	720	2.5
5	A	Farmland	10.0	82.0	0.24	0.22	1.15	810	3.2
6	B	Coastal	4.5	72.0	0.58	0.19	1.05	550	1.8
7	B	Coastal	6.5	75.0	0.45	0.24	1.21	680	2.4
8	B	Coastal	10.0	82.0	0.25	0.31	1.42	830	3.5
9	B	Industrial	3.0	71.5	0.61	0.21	1.12	520	1.5
10	B	Industrial	5.5	74.8	0.48	0.27	1.30	650	2.2
11	B	Industrial	8.5	80.2	0.29	0.35	1.55	790	3.1
12	B	Mixed	2.5	70.2	0.68	0.13	0.85	450	1.3
13	B	Mixed	5.0	73.9	0.50	0.18	1.08	580	1.9
14	B	Mixed	7.5	77.6	0.37	0.24	1.26	700	2.6
15	B	Mixed	9.0	80.8	0.27	0.29	1.40	780	3.0

**Table 5:** Prediction results and errors for the 15 independent validation samples.

Sample No.	Manufacturer	Environment Type	Actual Service Time (Year)	Predicted Service Time (Year)	Absolute Error (Year)	Relative Error (%)
1	A	Farmland	2	2.4	0.4	20
2	A	Farmland	4	3.4	0.6	15
3	A	Farmland	6	6.5	0.5	8.3
4	A	Farmland	8	7.1	0.9	11.3
5	A	Farmland	10	10.3	0.3	3
6	B	Coastal	4.5	5.3	0.8	17.8
7	B	Coastal	6.5	7.6	1.1	16.9
8	B	Coastal	10	11.2	1.2	12
9	B	Industrial	3	2.1	0.9	30
10	B	Industrial	5.5	6.4	0.9	16.4
11	B	Industrial	8.5	9.7	1.2	14.1
12	B	Mixed	2.5	3.2	0.7	28
13	B	Mixed	5	5.8	0.8	16
14	B	Mixed	7.5	8.3	0.8	10.7
15	B	Mixed	9	10.1	1.1	12.2
Average (Same manufacturer)	-	-	-	-	0.55	13.5
Average (Cross manufacturer)	-	-	-	-	1	17.1

**Table 6:** Summary of validation accuracy and confidence-interval analysis.

95% CI/Prediction-Interval Result	RMSE (Year)	MAE (Year)	Samples	Validation Item
MAE 95% CI: 0.39–0.71	0.64	0.55	n = 5	Same-manufacturer hold-out test
MAE 95% CI: 0.74–1.26	1.16	1.00	n = 10	Cross-manufacturer independent test
14/15 samples within 95% prediction interval	0.99	0.85	n = 15	Overall independent test
Validation R^2^ = 0.89 ± 0.03	0.78 ± 0.11	0.62 ± 0.08	45 samples; 5 folds	5-fold cross-validation

## Data Availability

The original contributions presented in this study are included in the article. Further inquiries can be directed at the corresponding author.
